# Formalin Evokes Calcium Transients from the Endoplasmatic Reticulum

**DOI:** 10.1371/journal.pone.0123762

**Published:** 2015-04-15

**Authors:** Michael J. M. Fischer, Kailey J. Soller, Susanne K. Sauer, Joanna Kalucka, Gianluigi Veglia, Peter W. Reeh

**Affiliations:** 1 Institute of Physiology and Pathophysiology, University of Erlangen-Nürnberg, Erlangen, Germany; 2 Department of Chemistry, University of Minnesota, Minneapolis, United States of America; 3 VIB Vesalius Research Center, Campus Gasthuisberg, KU Leuven, Leuven, Belgium; 4 Department of Biochemistry, Molecular Biology and Biophysics, University of Minnesota, Minneapolis, United States of America; University of Texas at Dallas, UNITED STATES

## Abstract

The formalin test is the most widely used behavioral screening test for analgesic compounds. The cellular mechanism of action of formaldehyde, inducing a typically biphasic pain-related behavior in rodents is addressed in this study. The chemoreceptor channel TRPA1 was suggested as primary transducer, but the high concentrations used in the formalin test elicit a similar response in TRPA1 wildtype and knockout animals. Here we show that formaldehyde evokes a dose-dependent calcium release from intracellular stores in mouse sensory neurons and primary keratinocytes as well as in non-neuronal cell lines, and independent of TRPA1. The source of calcium is the endoplasmatic reticulum and inhibition of the sarco/endoplasmic reticulum calcium-ATPase has a major contribution. This TRPA1-independent mechanism may underlie formaldehyde-induced pan-neuronal excitation and subsequent inflammation.

## Introduction

An subcutaneous injection of a 616 mM formaldehyde (5% formalin) solution into the forepaw of rats and cats induces a sustained pain-related behavior that can be scored by measuring lifting, licking, and flinching of the injected paw [1]. The consequences of variation in doses, point of injection and the scoring system have been studied extensively and reviewed [2]. The number of studies per year using the ‘formalin test’ rises continuously, its popularity stemming from the ease to perform a single intervention resulting in a reproducible ‘spontaneous’ pain-related response, which can be observed in an unrestrained animal.

We have recently demonstrated why two phases of activity, separated by a quiescent interphase, the hallmark of the test, come about [3]. Initially, formaldehyde excites the exposed sensory neurons, leading to the first phase of activity. Shortly thereafter, the formaldehyde-exposed neurons are hyperpolarized, leading to a silencing of these neurons before they are slowly but irreversibly inactivated by the high formaldehyde concentrations. The redistribution of the highly diffusible formaldehyde gas in the skin reaches and activates new surrounding skin areas, the basis of the second behavioral phase. The second activity phase fades when further redistribution and clearance lowers the concentration below the threshold of activation.

An open question is what formaldehyde does beyond the activation of the universal TRPA1 chemoreceptor channel [4]. A majority of the formaldehyde-induced effects on nociceptors was attributed to TRPA1, which is found primarily in the peripheral nervous system [5;6]. TRPA1 is activated by a variety of unsaturated electrophilic chemicals like allyl isothiocyanate and allicin, as well as by aldehydes (e.g. acetaldehyde and acrolein), including methylglyoxal which is increased in diabetic neuropathy patients [7–11]. The electrophilic chemicals covalently modify critical N-terminal intracellular cysteines [12], which includes formation of receptor-activating disulphide bridges for some of the agonists [7;13;14]. So far, TRPA1 was the only target of formaldehyde described to activate cells. Nonetheless, the ablation of the vast majority of nociceptive sensory neurons, including most of the neurons expressing TRPA1, has little effect on the biphasic formaldehyde-induced behavioral response if, as usual, a high concentration of formaldehyde (246 mM = 2%) is injected [15]. In contrast, pain-related behavior in response to an injection of 62 mM formaldehyde, just sufficient to induce an interphase and a second behavioral phase [3], is substantially reduced in TRPA1-deficient mice [4], indicating that 246 mM formaldehyde acts on an additional, yet unknown, molecular target. We identified a pan-cellular TRPA1-independent release of calcium from the endoplasmatic reticulum evoked by formaldehyde that may activate all cell types including sensory neurons.

## Materials and Methods

### Ethics statement

The use of animals was authorized by the animal protection authority of the district government ‘Veterinärmedizin und Verbraucherschutz—Sachgebiet 54, Regierung von Mittelfranken’ (Ansbach, Germany). All procedures were carried out in strict accordance to German legal regulations of animal care (TierSchG) and GV Solas (Society for laboratory animal science) recommendations.

### Animals

The study was performed using adult C57BL/6 mice of either sex. TRPA1^-/-^ animals were a generous gift from D. P. Corey [16]. All mice were bread in-house, including TRPA1^+/+^. Mice were housed in group cages in a temperature-controlled environment on a 12h light-dark cycle and were supplied with food and water *ad libitum*. Mice were sacrificed in a rising CO_2_ atmosphere.

### Substances and solutions

Formaldehyde was bought as a 37% solution (formaldehyde saturated solution, assured concentration 37–37.5 mass%, stabilized by methanol 8–12%, Carl Roth, Karlsruhe, Germany). Formaldehyde was prepared in ultrapure water and freshly diluted in extracellular solution (in mM: 140 NaCl, 5 KCl, 2 CaCl2, 2 MgCl2, 10 HEPES, 10 glucose, pH 7.4). To avoid confusion between % of formalin and % of formaldehyde in water, the concentration of formaldehyde in mM is used throughout the manuscript. Ionomycin, carbonyl cyanide m-chlorophenyl hydrazine (CCCP), A967079, BCTC, dantrolene and thapsigargin were obtained from Sigma-Aldrich, ryanodine and L-buthionine sulfoximine from Santa Cruz (Ann Arbor, MI).

### Cell Culture

Dorsal root ganglia (DRG) were harvested from the lumbar segments of adult C57BL/6 mice and Wistar rats and transferred to Dulbecco's modified Eagle's medium (Life Technologies, Germany) supplemented with gentamycin (50 μg/ml, Sigma-Aldrich, Germany). DRGs were incubated for one hour in 2.2 U/ml collagenase and 1.5 U/ml protease (Sigma-Aldrich). After enzymatic digestion, the suspension was dissociated with fire-polished Pasteur pipettes and plated on glass cover slips coated with Poly-D-Lysine (200 μg/ml, Sigma-Aldrich). DRG neurons were incubated in serum-free TNB 100 medium supplemented with TNB 100 protein-lipid complex (Biochrom, Berlin, Germany), penicillin and streptomycin (100 U/ml each, Life Technologies, Germany) and nerve growth factor (mouse NGF 2.5S, 100 ng/ml; Alomone Labs, Tel Aviv, Israel) at 37°C and 5% CO_2_. Electrophysiological recordings or calcium-imaging were performed within 15–30 hours after dissociation. Primary mouse keratinocytes were isolated from skin of newborn mice as described before [17]. Briefly, the skin was incubated with 250 mg/ml neutral protease (Dispase, Roche, Mannheim, Germany) overnight at 4°C. The epidermal layer was mechanically separated and incubated in Accutase (Sigma-Aldrich, Germany) for 15 min at room temperature. The resulting single-cell suspension was cultured in CnT57 medium (Cell-n-Tech, Bern, Switzerland) for 7 days, before reseeding in plastic dishes for optical measurement. HEK293t, ND7/23 and CHO-K1 cells were regularly passaged and plated for experimental use on glass cover slips coated with Poly-D-Lysine as mentioned above.

### Patch clamp recordings

Whole cell recordings in voltage clamp mode were performed on HEK293t cells. Membrane currents were acquired with an Axopatch 200B amplifier, controlled by pCLAMP software (Axon Instruments/Molecular Devices, Sunnyvale, CA). Data were filtered at 1 kHz and acquired at 2 kHz. Electrodes were pulled from borosilicate glass tubes (TW150F-3; World Precision Instruments, Berlin, Germany) and heat-polished to give a resistance of 2–3 MΩ. The standard external solution was mentioned above, the internal solution contained (in mM) 140 KCl, 2 MgCl_2_, 5 EGTA and 10 HEPES, pH 7.4 was adjusted with KOH. For voltage clamp recordings, cells were held at -60 mV. All recordings were performed at ~21°C room temperature. Solutions were applied with a gravity-driven system.

### Intracellular ratiometric calcium measurements

DRG neurons were loaded with the fluorescent calcium indicator dye Fura-2-AM 3 μM supplied with 0.02% pluronic F-127 (Invitrogen Molecular Probes, Eugene, OR, USA) for 30 min. Fura-2 was excited at 340 and 380 nm with 3 ms exposure time at 1 Hz, fluorescence at >490 nm was collected. For measurements of the keratinocytes on plastic dishes, a ratio between the isosbestic wavelength of 358 nm and 391 nm was chosen; the latter showed the maximum relative change. Images were acquired with the TillVision software package from a peltier-cooled slow-scan CCD camera system with a PolyV monochromator (Till Photonics, Graefelfing, Germany) coupled to an inverted microscope. Cells were continuously superfused at a rate of 0.3 ml/min through a gravity driven and software-controlled common outlet perfusion system [18]. The extracellular solution contained 1.25 mM CaCl_2_ for microfluormetry, all substances were diluted in this solution. All experiments were performed at room temperature. The fluorescence ratio was calculated for all regions of interest after background subtraction. The AUC of the ratio during application periods compared to control periods was used for analysis. KCl 60 mM for neurons or ionomycin 2 μM for cell lines was applied at the end of every experiment to discard non-responsive cells and acquire a maximum response.

### Sarco/endoplasmic reticulum Calcium-ATPase (SERCA) activity assays

SERCA1a extracted from rabbit skeletal muscle and purified by reactive red affinity chromatography as previously described [19]. SERCA was reconstituted into 4:1 DOPC:DOPE lipid vesicles at a 1:700 SERCA:lipid ratio [20;21] and activity was measured using a coupled enzyme assay [22]. SERCA was incubated with formaldehyde for 30 minutes before starting the assay. NADH consumption at 340 nm was monitored with a Spectromax microplate reader (Molecular Devices, Sunnyvale, CA) to give initial rates of SERCA activity as a function of calcium concentration at 37°C. These initial rates were plotted versus calcium concentration and the data were fit to the Hill equation to obtain V_max_.

$$V=\frac{V_{\max}}{1+10^{n(pK_{Ca}-pCa)}}$$

### Data analysis

Statistical comparisons were performed with Statistica (Statsoft, Tulsa, USA). Results were tested with a matched pairs t-test. p < 0.05 was considered to be significant. A sigmoidal function was fitted to concentration-response data using Origin (OriginLab Corporation, Northampton, USA). Data are presented as mean ± SEM.

## Results

### TRPA1-independent formaldehyde responses in cultured cell lines

**Fig 1:**
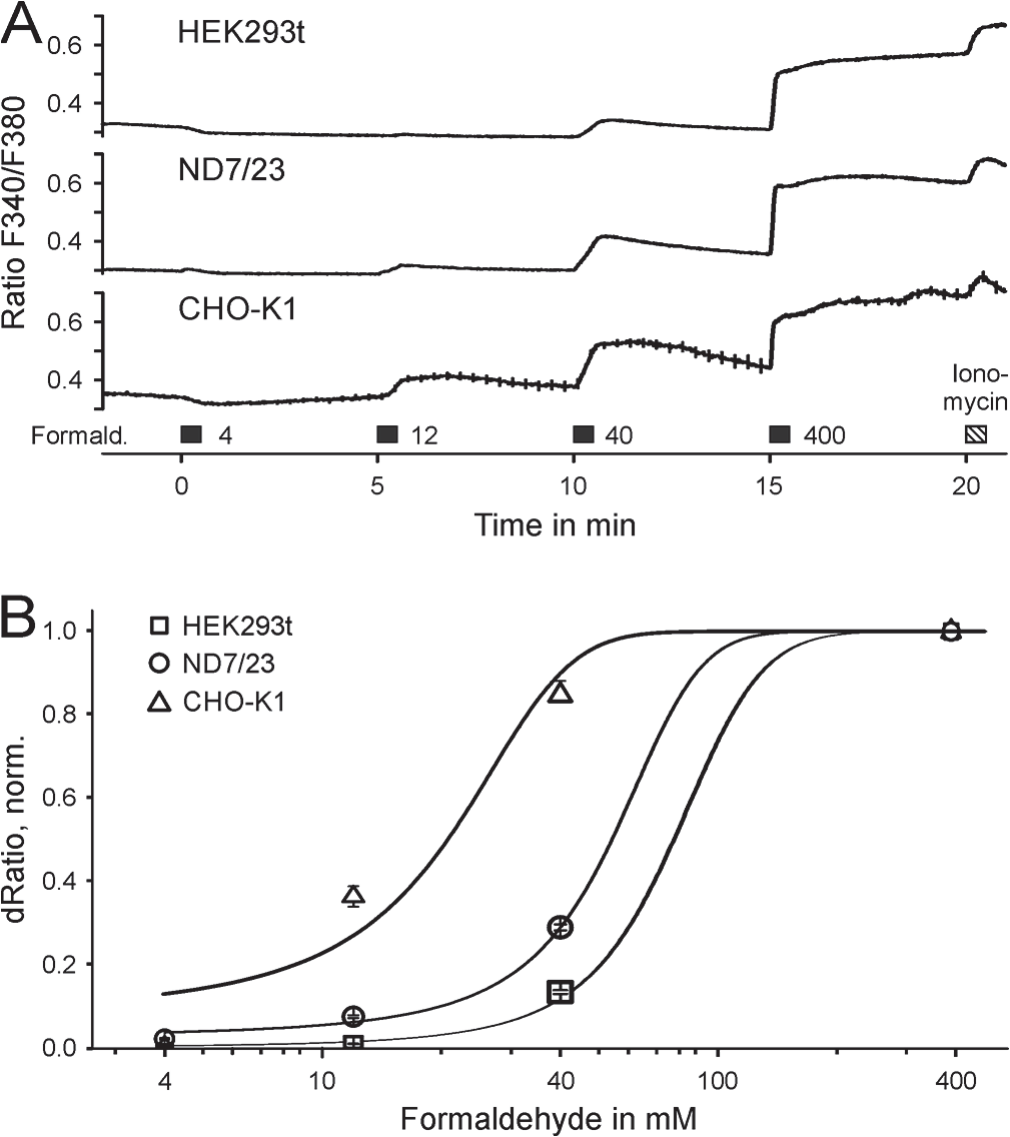
Formaldehyde-induced calcium release in cell lines. A) Cells of common cell lines were exposed to increasing concentrations of formaldehyde. Chinese hamster ovary (CHO-K1, n = 58) cells were more sensitive to formaldehyde than a mouse neuroblastoma rat neuron hybrid cell line (ND7/23, n = 370) and HEK293t cells (n = 276). Responses in CHO-K1 cells were similar compared to DRG neurons [3]. Formaldehyde concentrations up to 40 mM show slowly reversible responses. B) Responses are normalized to formaldehyde 400 mM, which causes a permanent submaximal calcium increase. Note that in the original formalin test, 616 mM are injected into the paw.

To investigate the mechanism of TRPA1-independent activation we employed cell lines. HEK293t, CHO-K1 and ND7/23 were exposed to formaldehyde; sensitivity to respond with a calcium increase was markedly different in the order CHO-K1 > ND7/23 > HEK293t, apparent at 12 and 40 mM formaldehyde (ANOVA, F_(2,700)_ = 693, p < 0.001 between pairs of cell lines, Fig 1A). The respective half-maximal effective concentrations were 21 ± 5 mM, 54 ± 2 mM and 69 ± 2 mM. We have previously shown that responses in HEK293t cells show a similar concentration-response as TRPA1^-/-^ DRG neurons [3]. Formaldehyde at a concentration up to 40 mM elicited a reversible response, whereas 400 mM lead to a persistent calcium increase. Antagonists for TRPA1 and TRPV1 did not alter this outlasting formaldehyde-induced calcium increase (S1 Fig). Depletion of glutathione levels in HEK293t cells also did not alter the response to formaldehyde (S2 Fig).

**Fig 2:**
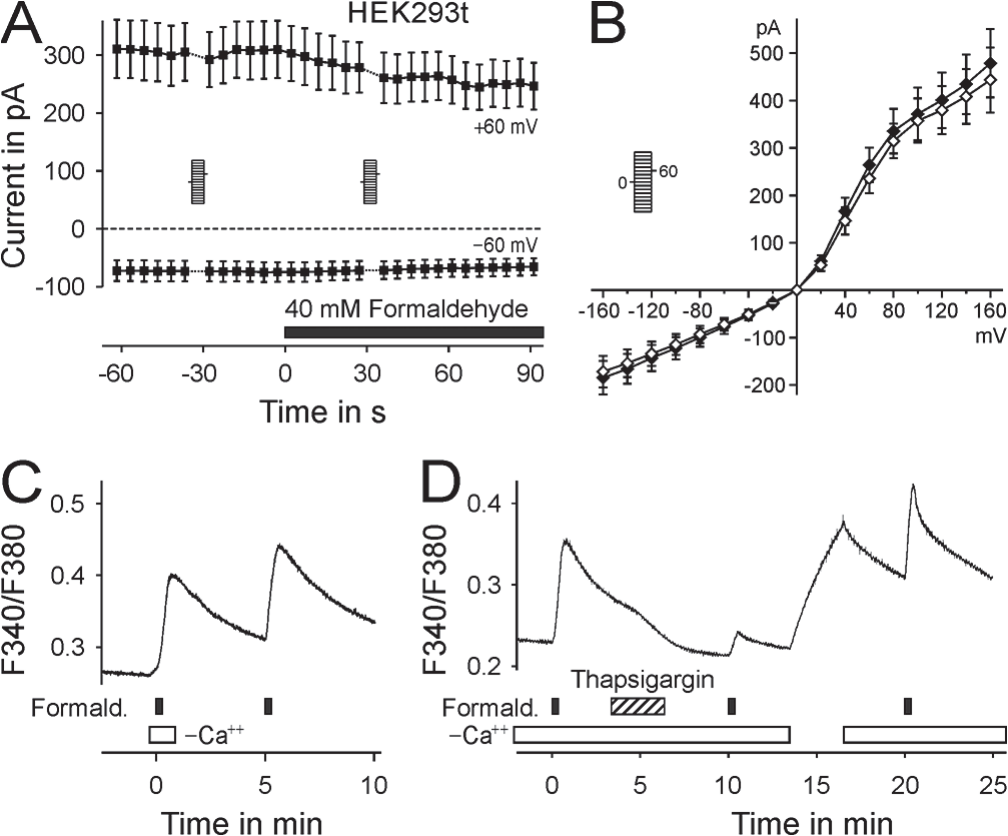
Formaldehyde releases calcium from intracellular stores in untransfected HEK293t cells. A) HEK293t cells were patch-clamped at—60 mV, voltage ramps were applied at an interval of 5 seconds. The currents at ±60 mV of these voltage ramps are displayed; exposure to formaldehyde did not change transmembrane currents (n = 9). B) The steady-state current-voltage relationships 30 s before (open symbols) and during (closed symbols) exposure to formaldehyde are not significantly different (n = 12). C) Calcium transients evoked by formaldehyde 40 mM in HEK293t cells (n = 235) are similar in absence and presence of extracellular calcium, indicating an intracellular release and explaining the lack of a detectable transmembrane current. D) Calcium transients evoked by formaldehyde 40 mM are decreased after exposure to Thapsigargin 5 μM. Formaldehyde responses recovered after replenishing intracellular calcium stores (n = 111). Note the slight delay in calcium clearance due to the application of Thapsigargin, indicating a slow calcium efflux out of the stores when SERCA is inhibited.

To investigate the source of this calcium transient, whole-cell transmembrane currents in HEK293t cells were investigated. Currents at positive and negative holding potentials were unchanged by exposure to formaldehyde 40 mM, indicating no effects at plasma membrane ion channels (Fig 2A). The current-voltage relationship 30 seconds after exposure to formaldehyde, corresponding to the peak of the calcium increase, was unchanged as compared with before exposure to formaldehyde (Fig 2B). This indicated release from an intracellular calcium source, which was confirmed in HEK293t cells by a similar calcium increase in extracellular solution without and with calcium (dRatio 0.102 and 0.108, n = 235, Fig 2C). In the absence of extracellular calcium, depletion of endoplasmatic reticulum calcium stores through a brief three minutes application of thapsigargin 5μM substantially reduced the second response to formaldehyde to 28% of the first response (p < 0.001, n = 111, t-test compared to second application, Fig 2D). After supplying extracellular calcium for three minutes the formaldehyde response recovered to 126% of the control (p < 0.001, t-test compared to second application).

**Fig 3:**
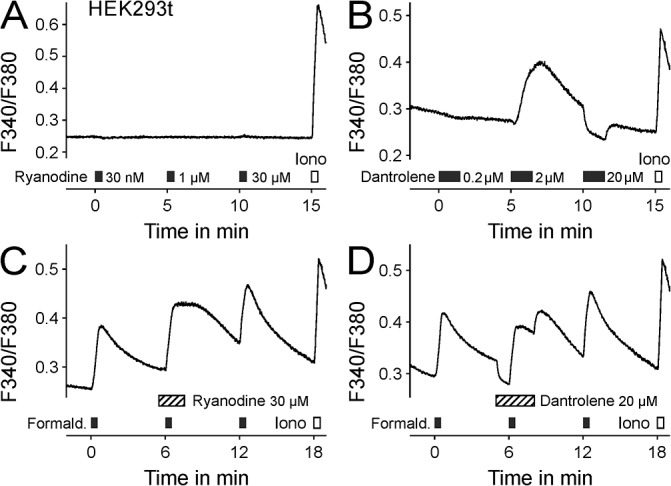
Formaldehyde does not release calcium in HEK293t via ryanodine or IP3 receptor activation. A) In HEK293t cells no responses were induced by ryanodine at the given concentrations. B) Dantrolene increased intracellular calcium at 2 μM, but reduced calcium levels at 20 μM. C) The magnitude of repetitive formaldehyde-induced calcium responses was not altered by co-administration of ryanodine 30 μM or D) dantrolene 20 μM.

For further mechanistic insight how the endoplasmatic reticulum calcium stores are affected, we tested whether formaldehyde alters the ryanodine or IP3 receptor function. We did not observe any calcium release in response to ryanodine at increasing concentrations (Fig 3A). Dantrolene 2 μM increased intracellular calcium, but at an IP3 receptor-inhibiting concentration of 20 μM [23], calcium levels were reduced (Fig 3B). Repetitive formaldehyde applications induced calcium increases of similar magnitude, irrespective of ryanodine or dantrolene coapplications (Fig 3C and 3D).

### TRPA1-independent formaldehyde responses in keratinocytes and sensory neurons

**Fig 4:**
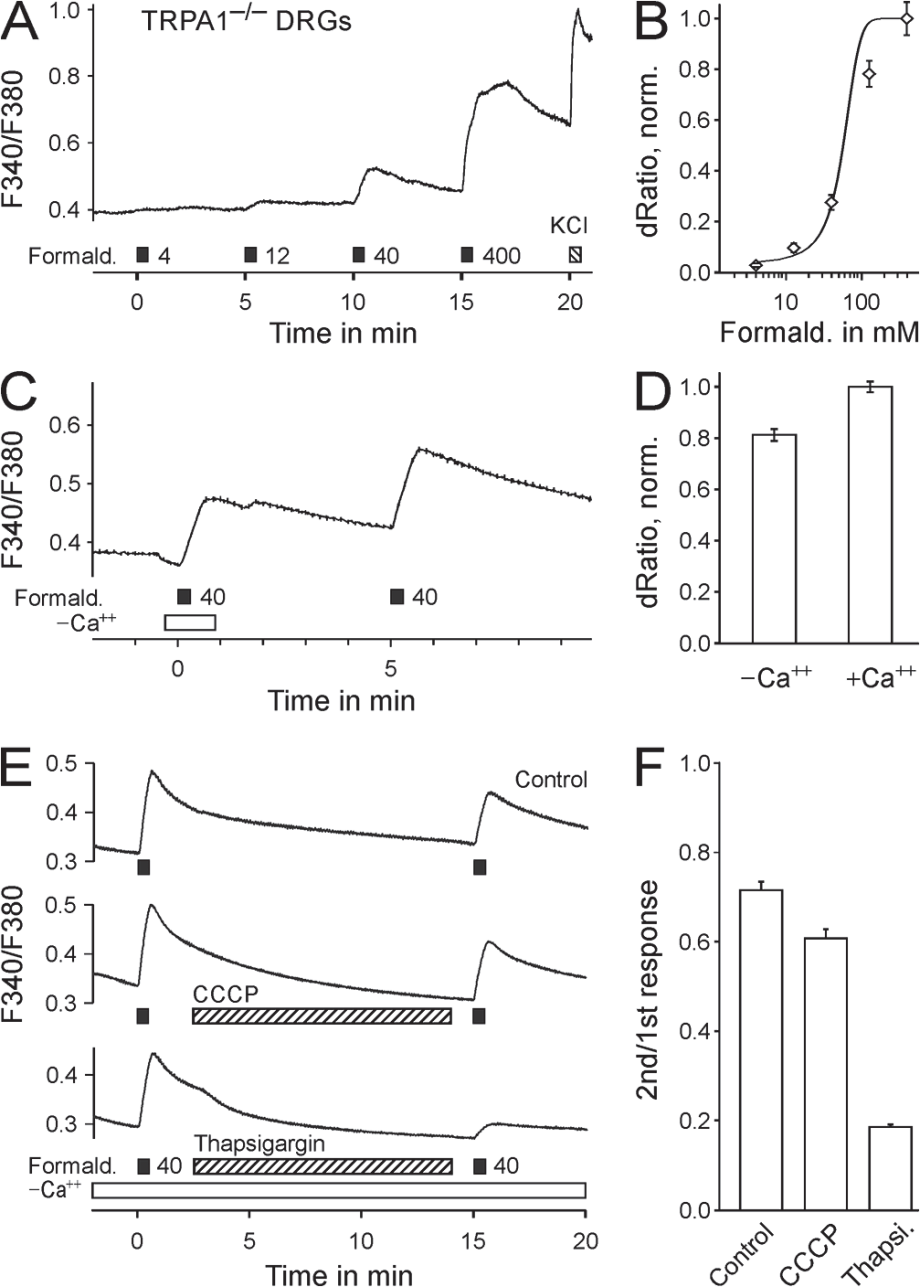
Formaldehyde activates TRPA1-deficient DRG neurons by release of calcium from the endoplasmatic reticulum. A) Formaldehyde increases intracellular calcium levels in cultured TRPA1^-/-^ DRG neurons. B) Concentration-response of calcium transients, normalized to formaldehyde 400 mM (n = 71). A second protocol including formaldehyde 126 mM (not shown) provided an additional data point for a robust concentration-response fit. C) In the absence of extracellular calcium formaldehyde 40 mM induced intracellular calcium increases in DRG neurons from TRPA1^-/-^ mice (n = 212). D) Calcium transients in the absence of extracellular calcium were 81% of the subsequent response in the presence of extracellular calcium. E) In the absence of extracellular calcium, formaldehyde was applied twice (Control, no application during the period indicated by the hatched bar, n = 143). In experiments depleting mitochondrial calcium stores (hatched bar: incubation with CCCP 2 μM, n = 147), the responses induced by formaldehyde had a similar magnitude as the control. However, if the calcium stores of the endoplasmatic reticulum were depleted by SERCA inhibition (hatched bar: Thapsigargin 2 μM, n = 148), the second response was reduced to 19% of the first response. F) Second formaldehyde-induced calcium responses of TRPA1^-/-^ neurons normalized to the first response.

**Fig 5:**
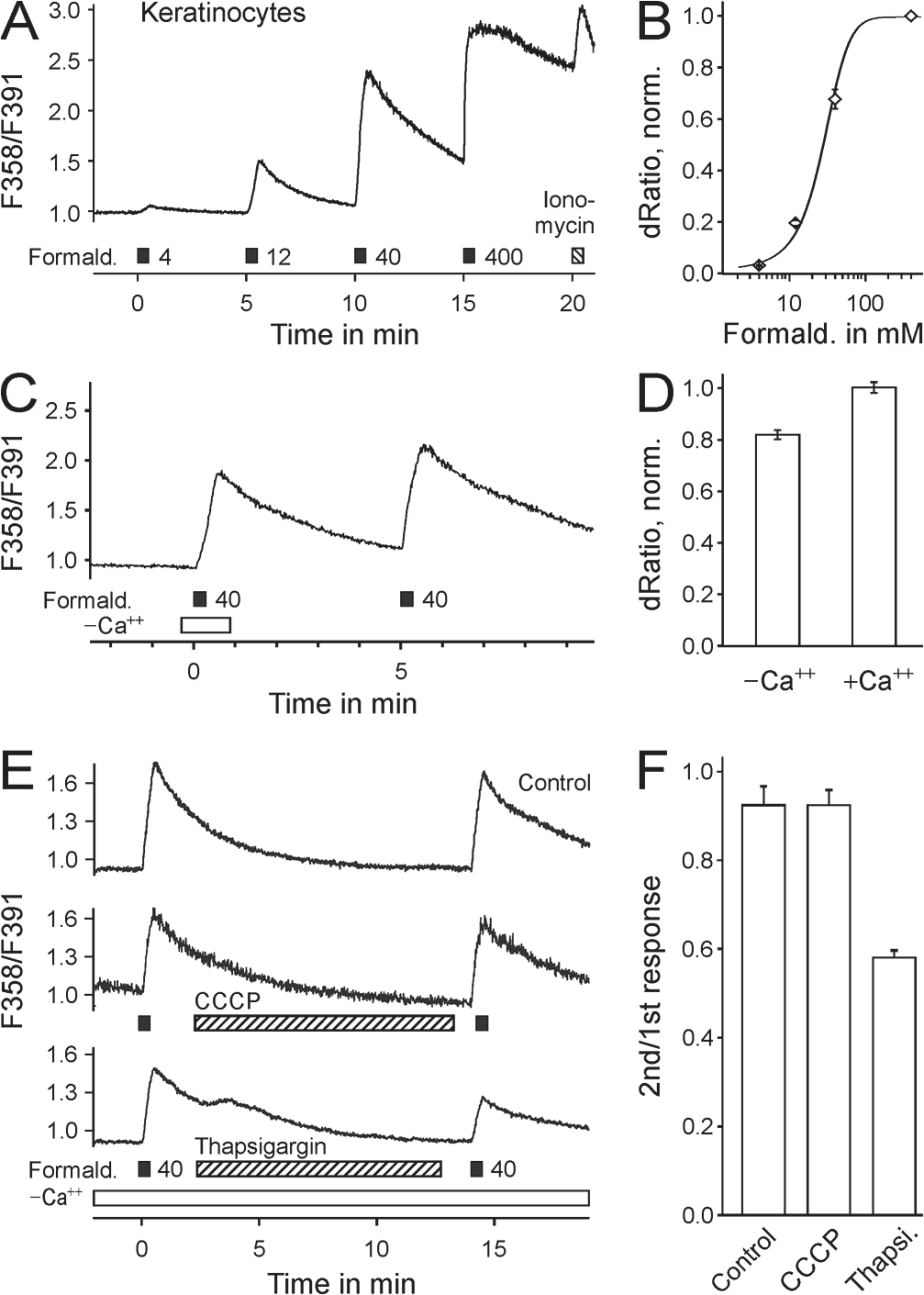
Formaldehyde activates mouse keratinocytes by release of calcium from the endoplasmatic reticulum. A) Formaldehyde increases intracellular calcium levels in primary cultured C57BL/6 keratinocytes. B) Concentration-response of calcium transients, normalized to formaldehyde 400 mM (n = 142). C) In the absence of extracellular calcium formaldehyde 40 mM induced intracellular calcium increases in mouse keratinocytes (n = 170). D) Calcium transients in the absence of extracellular calcium were 82% of the subsequent response in the presence of extracellular calcium. E) In the absence of extracellular calcium, formaldehyde was applied twice (Control, no application during period indicated by the hatched bar, n = 165). In experiments depleting mitochondrial calcium stores (hatched bar: incubation with CCCP 2 μM, n = 119) the responses induced by formaldehyde had a similar magnitude as the control. However, when the calcium stores of the endoplasmatic reticulum were depleted by SERCA inhibition (hatched bar: thapsigargin 2 μM, n = 181), the second response was reduced to 58% of the first response. F) Amplitude of the second formaldehyde-induced calcium responses of keratinocytes, normalized to the first response.

Injection of formaldehyde into the hind limb acts on all cell types present in the skin. Cultured sensory neurons as well as cultured keratinocytes of C57BL/6 mice were exposed to increasing formaldehyde concentrations. The evoked intracellular calcium increase had an EC_50_ of 57 ± 11 mM in TRPA1^-/-^ sensory neurons (n = 217, Fig 4A and 4B) and 29 ± 4 mM in wildtype keratinocytes (n = 217, Fig 5A and 5B). In the absence of extracellular calcium formaldehyde 40 mM elevated intracellular calcium levels in cultured dorsal root ganglion neurons from TRPA1^-/-^ mice (dRatio without calcium 0.086 compared to 0.106 with calcium, n = 212, Fig 4C) as well as in cultured keratinocytes (0.70 without calcium compared to 0.86 with calcium, n = 170, Fig 5C).

To identify the source of formaldehyde-stimulated cytoplasmatic calcium increases, repeated formaldehyde 40 mM stimulation was performed in the absence of extracellular calcium. In control experiments in TRPA1^-/-^ DRG neurons, the second formaldehyde stimulation increased calcium by 72% of the response to the first stimulation (n = 143, Fig 4E). When mitochondrial calcium levels were depleted by uncoupling the mitochondrial proton-gradient with CCCP 2 μM, the second response was 61% of the first response (p = 0.57, n = 147, t-test with independent samples vs. experiment without CCCP). However, when endoplasmatic reticulum calcium stores were depleted with the SERCA inhibitor thapsigargin 5 μM, the second response was 19% of the first response (p < 0.001, n = 148, t-test with independent samples, Fig 4F). Similar, in keratinocytes the second response to formaldehyde was 94% of the first response in control experiments (n = 165), 92% after exposure to CCCP 2 μM (p < 0.68, n = 119, t-test with independent samples vs. experiment without CCCP), and substantially lower at 58% after exposure to thapsigargin 2 μM (p < 0.001, n = 181, t-test with independent samples, Fig 5E and 5F).

### Formaldehyde inhibits SERCA’s ability to transport calcium into the endoplasmic reticulum

**Fig 6:**
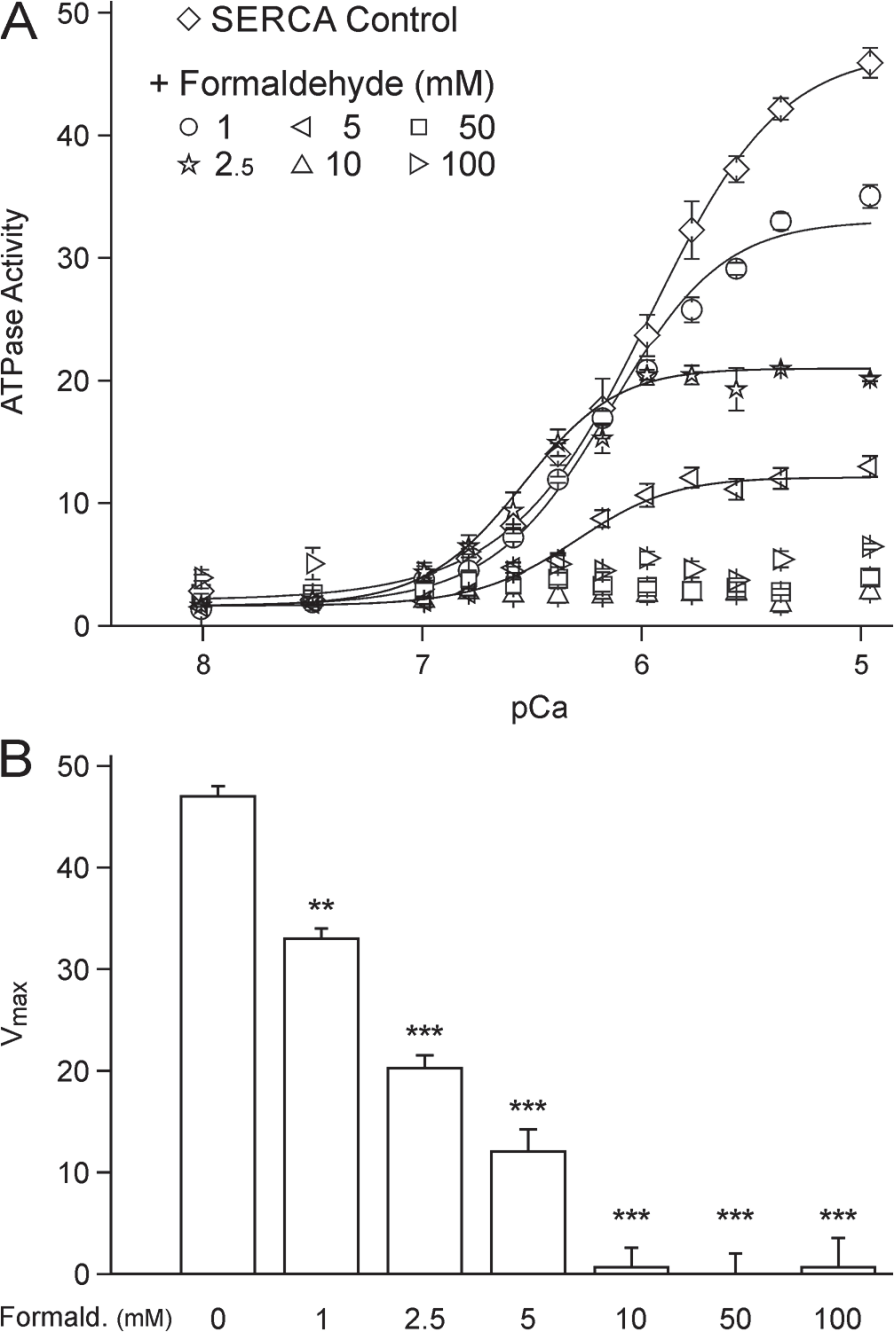
Formaldehyde impairs SERCA activity. A) SERCA activity is impaired by pre-exposure to formaldehyde for 30 min as measured by NADH-coupled enzyme assay (n = 3 for each point). Activity as a function of the calcium concentration (negative logarithm pCa) was fit using the Hill equation. B) V_max_ was decreased to 70% of the initial SERCA control value upon pretreatment by 1 mM formaldehyde and SERCA activity was abolished by ≥10 mM formaldehyde. *p < 0.05, and ***p < 0.001.

The effects of formaldehyde pretreatment on the activity of the SERCA was measured at several formaldehyde concentrations as a function of different calcium concentrations at 37°C. Formaldehyde was added to the reconstituted SERCA system at final concentrations of 1, 10, 50 and 100 mM formaldehyde (n = 3 for each point). The activity of SERCA alone and SERCA with 1 mM formaldehyde can be fit extremely well with the Hill equation, with R^2^ values over 0.99. With the addition of 1 mM formaldehyde, only V_max_ is decreased to 70% of SERCA’s V_max_ alone (p < 0.027, n = 3, Fig 6A and 6B). At ≥10 mM formaldehyde the effective SERCA activity was no longer quantifiable and the SERCA activity reduced to the point where the Hill function did not reliably fit the data.

## Discussion

Formaldehyde concentrations as used in the common formalin test release calcium from intracellular stores. This effect is reduced after calcium depletion of the endoplasmatic reticulum but not of the mitochondria. Pharmacological inhibition and direct measurement of enzyme activity indicate the SERCA a principal target.

Formaldehyde activated TRPA1 with an EC50 of 2.5 μM but also evoked TRPA1-independent effects with an EC50 of 14.9 μM [3]. The original ‘formalin test’ uses 616 mM formaldehyde, saturating both the TRPA1-dependent and-independent mechanism in the first phase of the behavioral response. Formaldehyde concentrations above 30 mM have a considerable persistent inactivating component [3], therefore lower concentrations used in vitro overestimate the TRPA1-dependent and underestimate the TRPA1-independent component. Combined genetic and pharmacological ablation of the vast majority of afferent C-fibers (TRPV1-expressing neurons, MrgprD-expressing neurons or both of these populations), including those expressing TRPA1, reduced pain-related behavior in response to injection of 62 mM formaldehyde (0.5% formalin), but had little effect when in case 246 mM formaldehyde was used [15].

Our recordings support the notion that formaldehyde activates a variety of cell types including nerve fibers other than those expressing TRPA1. Analysis of DRG neurons, including TRPA1 deficient ones, showed a calcium response to formaldehyde 40 mM in every cell. Unexpectedly, these responses persisted in the absence of extracellular calcium and are therefore attributed to a release of calcium from an intracellular source. This suggests an ubiquitously expressed target of formaldehyde responsible for the TRPA1-independent effects. A TRPA1-independent formaldehyde activation was confirmed by the calcium transients observed in native cell lines. CHO-K1 and ND7/23 cells turned out to be more sensitive to formaldehyde compared to HEK293t cells, and the variation among the chosen cell lines was as much as the difference between TRPA1 wildtype and knockout DRG neurons (S2B Fig). In HEK293t cells, DRG neurons and keratinocytes formaldehyde released calcium from intracellular stores, i.e. in the absence of extracellular calcium. Pharmacological experiments with thapsigargin and CCCP indicate the endoplasmatic reticulum but not the mitochondria as the source of the cytoplasmatic calcium rise.

Two cell types, keratinocytes and sensory neurons, are first to be affected by the injection in the formalin test. As several cell lines responded to formaldehyde, we investigated keratinocytes in primary cell culture which also showed similar calcium transients from intracellular sources. Also for other irritants, a calcium elevation in keratinocytes has been demonstrated [24], also via TRPV1 and TRPA1 channels [25]. It has been shown that keratinocytes can release several messengers, including ATP, PGE_2_, LTE_4_ and NGF [25;26], which can increase neuronal excitability [27]. A direct calcium-dependent increase in excitability of neurons has been observed [28], however, also the contrary [29]. The contribution of calcium to excitability by the standard Hodgkin-Huxley model has also been explained by a shift in the so-called ‘FitzHugh-Nagumo phase portrait’ [30]. This might also involve the activation or sensitization of ion channels as it has been described e.g. for the calcium-gated chloride channel ANO1 [31]. The respective mechanisms have been reviewed [32], but are not fully resolved today.

In keratinocytes and neurons, finally, the molecular target of the formaldehyde-induced calcium transients was addressed. Pharmacological results argue against an activation of ryanodine or IP3 receptors by formaldehyde, which leaves inhibition of the permanently active SERCA as most likely target. Using a liposomal reconstitution system for SERCA, an NADH-coupled enzyme assays showed that SERCA activity is completely abolished in the presence of formaldehyde ≥10 mM. Since SERCA is primarily responsible for transporting cytosolic calcium into the endoplasmatic reticulum, calcium accumulates in the cytosol if SERCA activity is diminished. The enzyme activity collapse is caused by formaldehyde concentrations between 1 mM and 10 mM which are slightly lower than the concentrations required for the calcium transients observed in the cultured cells. This is expected as the intracellular concentration resulting from extracellular exposure to formaldehyde is limited by the plasma membrane and therefore lower compared to the biochemical assay. Other targets of formaldehyde in the biochemical assay can be excluded as the reconstituted lipidic environment contains only SERCA and lipids. The reactive formaldehyde might have a similar effect on SERCA as hydrogen peroxide, which has been described to inhibit SERCA activity by modification of cysteine [33]. These experiments identify SERCA as a major target of formaldehyde, besides TRPA1 and sodium channels. Sodium channels are inhibited by formaldehyde [3;34]; besides, the electrical input resistance of the cell membrane rose during prolonged exposure to formaldehyde which indicated that major background leak currents are also affected [3].

It was shown that few residual nociceptive neurons are sufficient for a biphasic formalin test behavior to occur [15]. In any case, at least the first behavioral phase of the typical formalin test does not require TRPA1. The present work demonstrates calcium transients evoked by formaldehyde in various cell types including keratinocytes, which may release a large variety of different mediators to activate and/or sensitize primary sensory neurons. The relative importance of the cell types affected by the formalin test is unclear, a direct neuronal activation may occur as well as an indirect activation via exposed keratinocytes or other cells in the vicinity of nerve endings.

## Supporting Information

S1 Fig TRPA1 and TRPV1 do not contribute to the formaldehyde response. A) Repeated stimulation with formaldehyde caused two similar calcium transients, calcium levels largely recovered before the second stimulus (n = 103–147 per group). B) Application of antagonists for TRPV1 or TRPA1 did not inhibit the formaldehyde-induced calcium increase compared to the second stimulation.
(TIF)

S2 Fig Comparison of formaldehyde concentration-response curves. A) In order to deplete glutathione levels, HEK293t cells were exposed to the glutathione-gamma-synthase inhibitor L-buthionine sulfoximine (BSO). Concentration-response curves evoked by formaldehyde after exposure to BSO for 20 h were similar to control experiments (n = 200–233 per group). B) Concentration-response curves of cell lines (dashed lines, data as in Fig 1B) are compared with keratinocytes from C57BL/6 mice (dotted line, data as in Fig 5B), DRG neurons from TRPA1^-/-^ (solid line, data as in Fig 4B) and C57BL/6 mice (solid line, filled squares, n = 58). Wildtype DRGs, keratinocytes and CHO-K1 cells have as similar concentration-response, TRPA1^-/-^ DRGs, ND7/23 and HEK293t cells are slightly less sensitive.
(TIF)
