# Supplementary figures and images for: Formalin Evokes Calcium Transients from the Endoplasmatic Reticulum
Source: PLoS One. 2015 Apr 15;10(4):e0123762. doi: 10.1371/journal.pone.0123762 (PMC4398422; doi:10.1371/journal.pone.0123762)

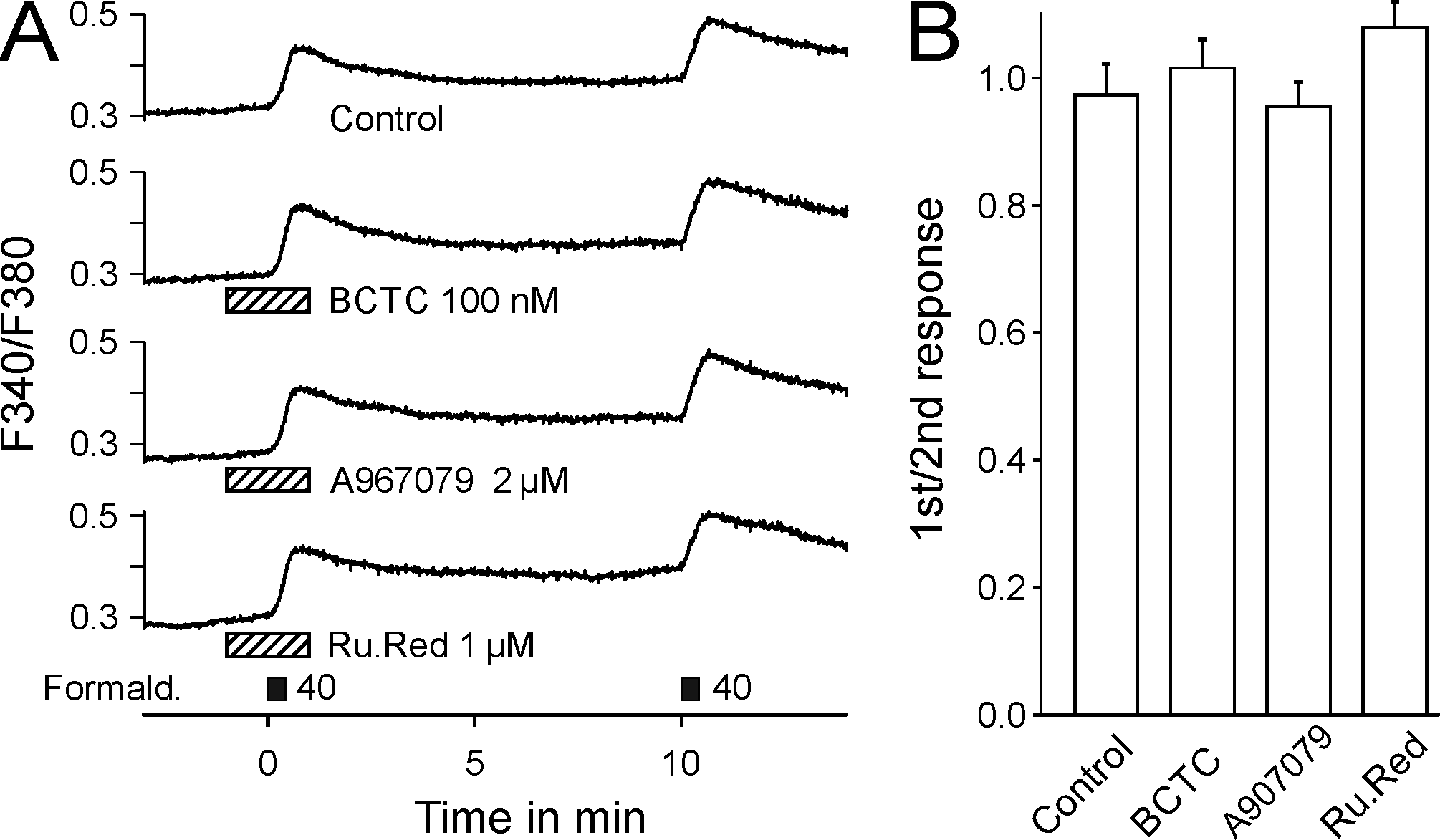

Supplement: S1 Fig — A) Repeated stimulation with formaldehyde caused two similar calcium transients, calcium levels largely recovered before the second stimulus (n = 103–147 per group). B) Application of antagonists for TRPV1 or TRPA1 did not inhibit the formaldehyde-induced calcium increase compared to the second stimulation. (TIF) [file pone.0123762.s001.tif]

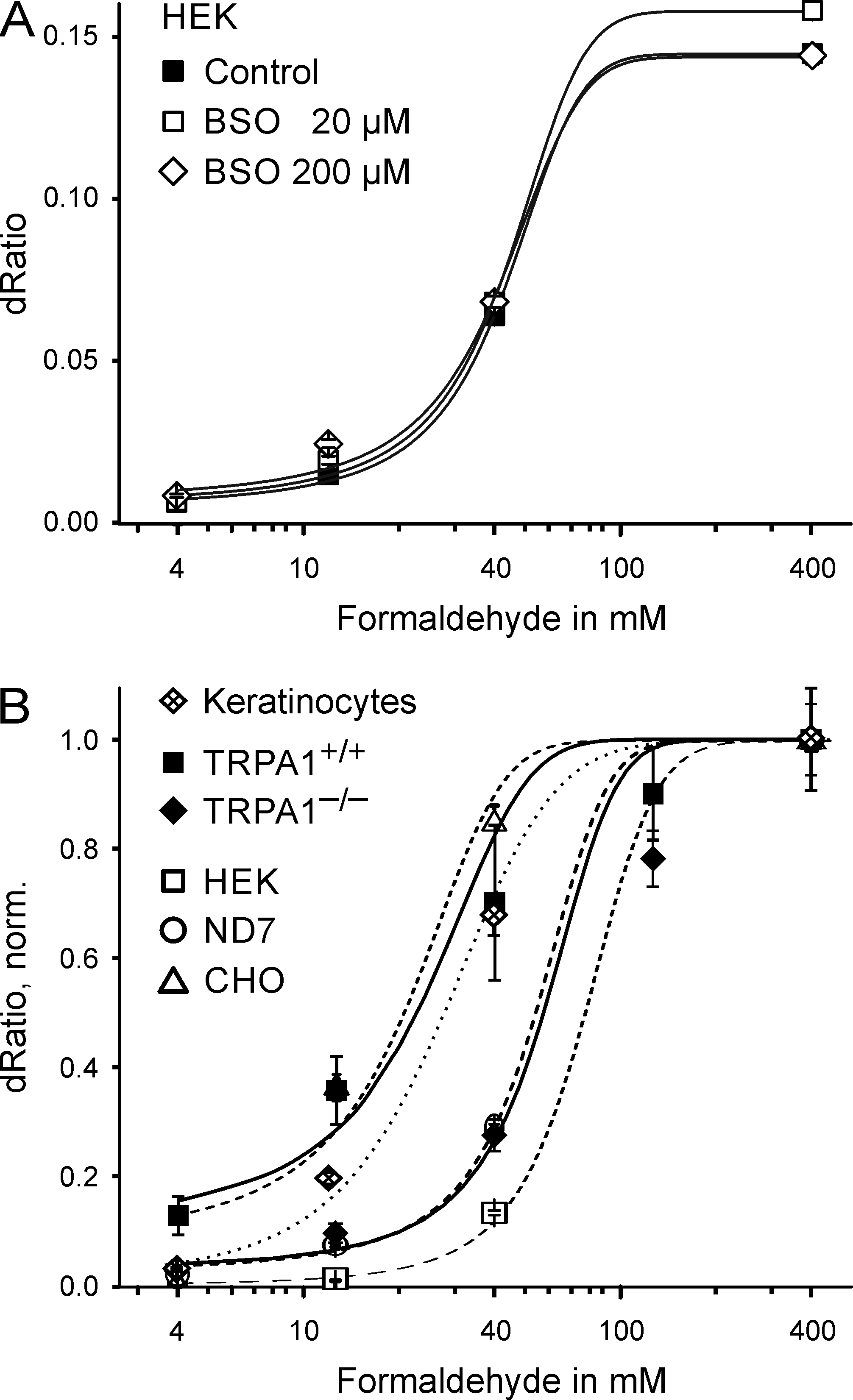

Supplement: S2 Fig — A) In order to deplete glutathione levels, HEK293t cells were exposed to the glutathione-gamma-synthase inhibitor L-buthionine sulfoximine (BSO). Concentration-response curves evoked by formaldehyde after exposure to BSO for 20 h were similar to control experiments (n = 200–233 per group). B) Concentration-response curves of cell lines (dashed lines, data as in Fig 1B) are compared with keratinocytes from C57BL/6 mice (dotted line, data as in Fig 5B), DRG neurons from TRPA1-/- (solid line, data as in Fig 4B) and C57BL/6 mice (solid line, filled squares, n = 58). Wildtype DRGs, keratinocytes and CHO-K1 cells have as similar concentration-response, TRPA1-/- DRGs, ND7/23 and HEK293t cells are slightly less sensitive. (TIF) [file pone.0123762.s002.tif]
